# Water-Assisted Production of Thermoplastic Nanocomposites: A Review

**DOI:** 10.3390/ma8010072

**Published:** 2014-12-29

**Authors:** József Karger-Kocsis, Ákos Kmetty, László Lendvai, Stavros X. Drakopoulos, Tamás Bárány

**Affiliations:** 1 Department of Polymer Engineering, Faculty of Mechanical Engineering, Budapest University of Technology and Economics, Műegyetem rkp. 3., Budapest H-1111, Hungary; E-Mails: karger@pt.bme.hu (J.K.-K.); kmetty@pt.bme.hu (A.K.); lendvai@pt.bme.hu (L.L.); 2 MTA-BME Research Group for Composite Science and Technology, Műegyetem rkp. 3., Budapest H-1111, Hungary; 3 Department of Material Science, University of Patras, Patras GR-26504, Greece; E-Mail: msci1347@upnet.gr

**Keywords:** nanocomposites, water-assisted melt compounding, clay, boehmite, commodity plastics, engineering thermoplastics, nanofillers, cellulose, thermoplastic starch

## Abstract

Water-assisted, or more generally liquid-mediated, melt compounding of nanocomposites is basically a combination of solution-assisted and traditional melt mixing methods. It is an emerging technique to overcome several disadvantages of the above two. Water or aqueous liquids with additives, do not work merely as temporary carrier materials of suitable nanofillers. During batchwise and continuous compounding, these liquids are fully or partly evaporated. In the latter case, the residual liquid is working as a plasticizer. This processing technique contributes to a better dispersion of the nanofillers and affects markedly the morphology and properties of the resulting nanocomposites. A survey is given below on the present praxis and possible future developments of water-assisted melt mixing techniques for the production of thermoplastic nanocomposites.

## 1. Introduction

Thermoplastic polymer nanocomposites have received considerable scientific and technological interest. This is reasoned by the fact that the properties of the corresponding polymer matrix can be prominently enhanced at a relatively low amount of nanofiller loading [1,2,3,4]. The nanocomposites may show improved mechanical, barrier, flame retardant, electrical and magnetic behaviors. Recently, research interest turned to ensure the composites with functional (conductivity, adaptive or stimulus-responsive features) rather than with structural (*i.e.*, mechanical) properties.

Thermoplastic nanocomposites are prepared by *in situ* polymerization, solvent-assisted techniques and melt mixing routes. Each of them has its advantages and disadvantages. As mentioned above, the water-assisted (WA) technique represents a combination of solution-assisted (mostly yielding the best dispersion) and traditional melt mixing (usually yielding the poorest nanofiller dispersion) methods [5]. Basic benefits of the WA technique are the followings:

- No need for surface modification of the nanofiller. This is especially important for such fillers, which should be rendered organophilic in order to achieve their acceptable dispersions. For suitable anionic and cationic clays ion exchange with suitable bulky surfactants is practiced for this purpose.
- No decomposition/degradation of the surface modifiers of the nanofillers as they are absent. This is a key issue for ammonium (onium) intercalated (surface modified) clays whose thermal decomposition limits the processing temperature and thus the possible range of polymers (they can never be used in high temperature resistant thermoplastics). It was found that the decomposition of alkyl ammonium ions starts as low as T = 180 °C though major thermal decomposition occurs between T = 200 °C and T = 500 °C [6]. It has to be born in mind that commodity and engineering thermoplastics are processed in the ranges of T = 190–250 °C and T = 200–290 °C, respectively.
- Reduced health risk when added in aqueous slurry compared to the dosage in dry powder form. This is a clear advantage when preformed (available *ob ovo* in nanoscale, such as silica, carbon nanotubes (CNT)) nanoparticles should be incorporated.
- Improved nanofiller dispersion due to local “blow-up” phenomena when the pressurized liquid evaporates from the melt. This was the basic idea of the early patent [7]. The other fundamental effects, linked with matrix/water (liquid) interactions include cryoscopy (*i.e.*, depression of the melting temperature associated with decreased melt viscosity) and plasticization. Attention should be called to the fact that some polymers are prone to hygrothermal decomposition. This manifests in substantially lower molecular weight (MW) products that should be counterbalanced in a proper way.
- There are further aspects worth mentioning. Water in some cases is an indispensable plasticizer that should not be removed during compounding. This is the case for the production of thermoplastic starch (TPS) from natural starch. So, gelatinization and nanoreinforcement of starch can be performed simultaneously [8]. In other cases, the liquid may work as reactive compound, for example for coupling molecular chains thereby enhancing the MW.
- Polymer and rubber lattices are aqueous dispersions, too. Like nanofiller dispersions these lattices can also be directly incorporated alone or in combination with suitable nanofillers to produce impact-modified (toughened) and nanoreinforced thermoplastic composites. This concept has been patented, as well [9,10].

The nanofillers can be grouped upon their origin, appearance, composition and the like. With respect to WA melt compounding the possible classification may consider the swelling ability, dispersibility. There are also several options for the matrix categorization. In our brief summary, the nanocomposites are grouped according to their matrices (commodity and engineering thermoplastics) and appearance of the nanofillers (quasi-spherical, disc-like or platy and needle-like or fibrous). Our intention is to survey next the research and development works making use of WA and liquid-assisted melt compounding techniques for the production of thermoplastic nanocomposites. Note that the production of nanocomposites may occur both discontinuously (batchwise, for example in an inner mixer) and continuously (extrusion melt compounding).

It is noteworthy that the dispersion spraying of nanofillers is a straightforward route also to create so called hierarchical reinforcements and related composites [11]. Under hierarchical reinforcement a peculiar combination of micro- (traditional fiber) and nanofiller (covering the microfiber) is meant [12].

## 2. Nanofillers

It is intuitive that suitable nanofillers should be water swellable or dispersible. Many inorganic and organic fillers may meet these requirements. Next they will be classified as quasi-spherical (termed to as zero-dimensional, 0D), fibrous (1D) and platy or disc-like (2D). Here is the right place to mention that 3D nanofillers do exist. One has to consider the nanoporous frameworks of zeolites (natural) and molecular sieves (synthetic). In these cases, however, the porous structure should be infiltrated by the polymers (eventually formed *in situ*), for which the WA technique is less suited.

Among the 0D nanofillers, silica, TiO_2_, Al_2_O_3_ should be explicitly mentioned. They are often surface treated for better dispersibility in aqueous media. On the other hand, these quasi-spherical nanofillers, having low aspect ratio, are rarely used in WA melt compounding—except boehmite alumina (BA) that is introduced later. Possible reason behind this fact is that these nanofillers are available in surface treated forms, tailored for good dispersion in given polymers.

Micro- and nano-fibrillated cellulose, carbon nanotube (CNT), carbon nanofiber (CNF) and halloysite nanotubes should be listed among the 1D or fibrous nanofillers. Note that their length to thickness (aspect) ratio may be very high (several thousands). Nanocellulosic fibrils are obtained from cellulosic fibers via chemical or enzymatic treatments combined with further disintegration processes. Due to the many hydroxyl groups (six per cellulose unit), the cellulose micro- and nanofibrils can be suspended in water well [13]. The dispersion of single-, double-, and multi-walled CNTs (SW-CNT, DW-CNT, and MW-CNT, respectively), produced usually by chemical vapor deposition, in aqueous fluids is a challenging task. Nevertheless, this can be achieved by using suitable surfactants, or by surface modification of the CNTs. The van der Waals interactions between the entangled CNTs can be overcome by input of mechanical energy (generally in the form of sonication) and surfactant molecules absorbing onto the surface of exfoliated CNT walls. For this absorption π(CNT)-cation(surfactant) interactions may be exploited [14]. The dispersion stability is guaranteed by electrostatic and/or steric repulsion [15]. The other possibility of rendering CNT hydrophilic is the generation of hydroxyl, carboxyl groups on their surfaces. This happens via wet chemistry, usually under the action of strong oxidizing acids. Researchers in this field usually follow versions of the Hummers-Offemann method [16]. Halloysite nanotubes belong to the family of aluminosilicates. They do not show strong tendency for agglomeration because few hydroxyl groups are located on their outer surfaces. The size ranges of the halloysite nanotubes are: inner diameter: 5–30 nm, outer diameter: 30–70 nm, and length: 0.1–15 μm, depending on the corresponding deposits [17].

**Figure 1:**
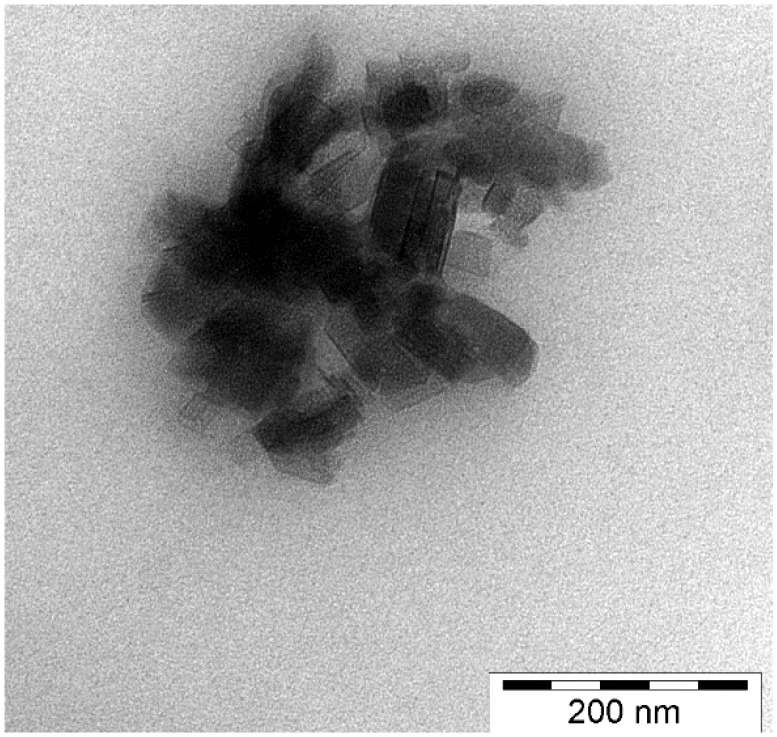
Transmission electron microscopic (TEM) picture showing Disperal^®^ 40 (Sasol, Germany) particles in a polyethylene (PE) matrix. The primary crystallite size of this BA is at about 40 nm.

Representative 2D nanofillers are layered silicates and graphene. Layered silicates of natural origin are generally termed as clays. Two to one layered silicates or phyllosilicates are most widely used for nanocomposite preparation. Their structure is a 2D sandwich in which one internal octahedral sheet (dominated by aluminum) is connected at the tip to two external tetrahedral silica sheets. The oxygen atoms of the latter belong to both sheets. When aluminum is substituted by other metal ions, the layers become negatively charged. This charge imbalance is compensated by cations (Na^+^, K^+^, Ca^2+^), absorbed between the trilayer sheets. The cations are held loosely and thus can be exchanged by other cations [1]. This is the basic principle of the organophilic modification during which the initial cations are replaced by bulky, organic cations. This expands the intergallery distance making it accessible for penetration of the polymer molecules. Many layered silicates (except some micas [18,19]) swell in water uptake thereby expanding the intergallery distance, similar to the organophilic modification. This is the reason why preferred nanofillers of WA are pristine cationic clays (montmorillonite, MMT and bentonite) as shown later. The aspect ratio of synthetic layered silicates may be as high as 6000 [19], by contrast to natural clays having an aspect ratio about 100 or less. Besides the above cationic clays, “anionic” versions also exist. Like cationic, the anionic clays may be of natural or synthetic origins. The structure of the related hydrotalcites, layered double hydroxides is, however, fundamentally different from phyllosilicates. The stacked crystal layers are composed of single octahedral metal hydroxide sheets. The excess positive charge of the layers is neutralized by interlayer anions. Though in the interlayer some water molecules may be present, these anionic clays swell in water only after suitable anion exchange [20,21]. This is the major reason why anionic clays are seldom used to produce nanocomposites in WA process. A further 2D nanofiller is graphite (when exfoliated but “stacked”), graphene (single atomic layer) and functionalized versions of the latter. Note that graphene is a 2D allotrope of carbon in atomic scale that is usually produced by top-down methods from layered graphite. To produce aqueous dispersions of graphene, similar strategies as discussed above for CNTs can be followed. Among the 2D nanofillers, we are listing boehmite alumina (BA) with the chemical structure of AlO(OH), *cf.* Figure 1. A peculiar feature of BA is that it may be classified into 0D, 1D and 2D nanofillers, because the required aspect ratios can be set by synthesis. Though BA can be found as a mineral as well, most of the research works used synthetic BA nanoparticles. They can be easily dispersed in water due to many hydroxyl groups on their surfaces.

To get information on the dispersion state of the nanofillers many different techniques can be used. Some of them are direct methods and adaptable for all nanocomposites, such as TEM, scanning electron microscopy (SEM), microcomputed tomography, atomic force microscopy. Others are also direct methods but usable only for given nanofillers, such as X-ray diffraction (XRD) to detect the intercalation/exfoliation of layered silicates. Among the indirect methods mechanical, rheological and permeability tests should be listed [22,23].

## 3. Concept and Realization of WA Melt Compounding

**Figure 2:**
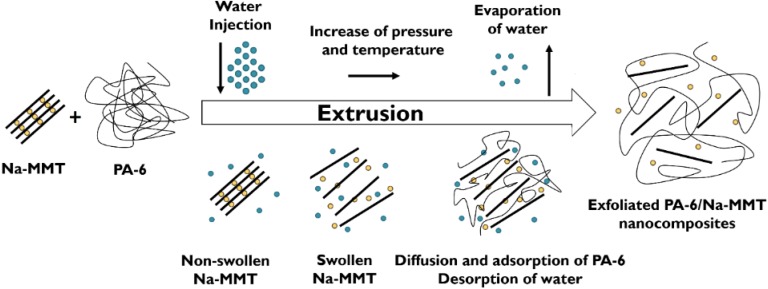
Montmorillonite (MMT) exfoliation in PA-6 during WA melt compounding schematically. Note: in this case water was injected into the molten PA-6 containing pristine MMT (Na-MMT).

The original concept was to inject water directly into the melt of polyamide-6 (PA-6) in the compression (high pressure) zone of the extruder, which was fed by PA-6 granules and natural clay [7]. Under high temperature and pressure, the water is fully miscible with PA-6, *i.e.*, PA-6 is soluble in water. Water acts not only as plasticizer (*i.e.*, reducing the glass transition temperature (T_g_)), but also as a melting temperature and crystallization suppressor [24,25]. This phenomenon is known under cryoscopic effect. Reduced melting temperature owing to cryoscopy is accompanied with lowered viscosity *per se* at a constant processing temperature. Accordingly, the polymer molecules are more mobile and even become more affine (*i.e.*, more polar) to the clay, which all favor the intercalation/exfoliation process of clay. Hasegawa *et al.* [26] modified the above outlined WA process by introducing the clay in aqueous slurry into the molten PA-6, but still in the high pressure compression zone of a special extruder. Dosage against high pressure requires adequate pumps and screw design, eventually with additional sealing rings, as well. In both above cases the water, as carrier of the clays, has been evaporated in the metering zone downstream of the extruder. Water removal was facilitated by suitably positioned vents (put also under vacuum). The intercalation/exfoliation of clay (mostly montmorillonite (MMT) types) occurred according to the scheme in Figure 2.

**Figure 3:**
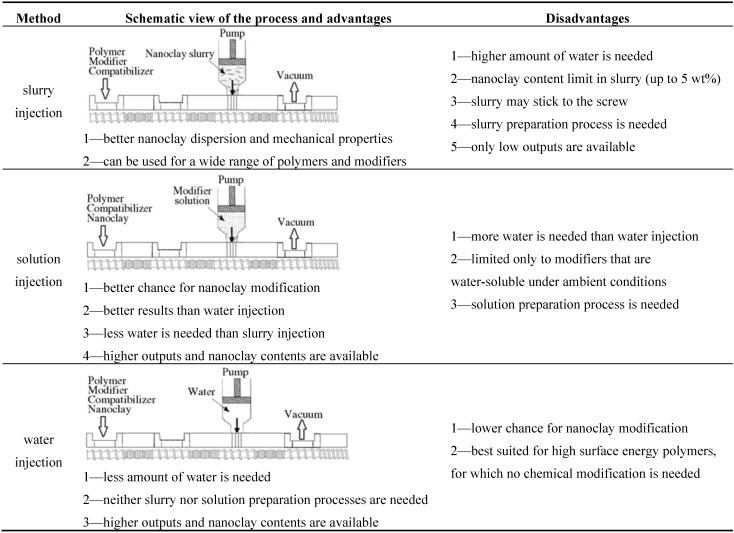
Advantages and disadvantages of various water-assisted (WA) melt compounding techniques introducing aqueous dispersion of the clay (“nanoclay”), aqueous solution of the modifier, or only water into the high pressure zone of an extruder (with permission of BME-PT) [30]. The “modifier” is a cationic surfactant which can replace the initial Na^+^ ions in between the clay layers.

As further milestone in the development of the WA strategy was the discovery that the organophilic modification of cationic clays can be done *in situ* during melt compounding of the polymer. This was demonstrated by Alexandre *et al.* [27]. In this pioneering work, pristine MMT (with Na^+^ cation, Na-MMT) was compounded with ethylene vinylacetate copolymer (EVA) in presence of dimethyl dioctadecylammonium bromide (cationic surfactant, modifier, intercalant). The latter surfactant has replaced the original Na^+^ cations in the MMT and facilitated the intercalation via the related clay gallery expansion in this “one pot reactive” process. It is noteworthy that the compounding was performed on an open two-roll mill, *i.e.*, in a discontinuous batch process. This development guided researchers to compare the efficiency of various WA techniques, namely (I) injection of water; (II) injection of aqueous solution of surfactants, and (III) injection of aqueous clay slurry into the molten polymer in the high pressure compression zone of the extruder. The above versions, along with their advantages and disadvantages, are displayed in Figure 3. Changing the matrix from a polar PA-6 to an apolar PE requested the use of a polymeric compatibilizer. For this purpose different co- and terpolymers and grafted polymers are mainly used, though maleic anhydride (MA) grafted homo- and copolymers belong to the present state of praxis. Compatibilizers act here mostly as “adhesives” creating a strong interphase between the polymer matrix and nanofiller particles.

**Figure 4:**
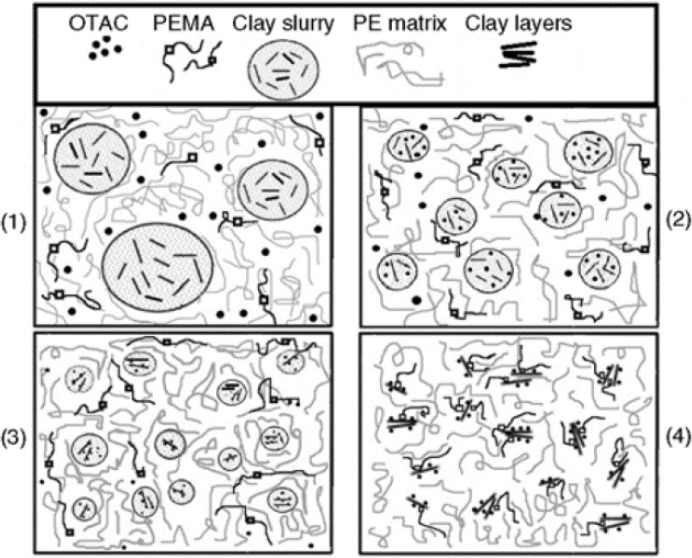
Schematic view of the intercalation mechanism in four steps in water-assisted (WA) melt compounding of polyethylene (PE) with clay slurry in presence of surfactant (OTAC) and compatibilizer (PEMA) (**1**) injection of clay slurry into the molten PE at high pressure; (**2**) OTAC is diffusing into the finer clay slurry drop and enter into cation exchange reaction; parallel to that water starts to evaporate; (**3**) water evaporation yields finer clay drops; cation exchange proceeds and compatibilizer works for uniform dispersion of the clay; (**4**) after complete evaporation of water the intercalates clay stacks and exfoliated layers are stabilized by OTAC and compatibilizer PEMA, especially when the latter contain coreactive groups (with permission of BME-PT) [30].

The authors have shown that the surfactant type and processing conditions (feeding rate, residence time) are controlling parameters of the clay dispersion. A further conclusion of the cited work was that the WA melt intercalation of PE is diffusion-controlled rather than shear-controlled for which a scheme was also proposed (*cf.* Figure 4).

The next milestone in the WA development can be traced to the discovery that the aqueous dispersion of the nanofillers can also be introduced in the pressureless feeding zone of the extruder. This method, pushed forward by the group of Karger-Kocsis [28], circumvents the use of high pressure pumps and special screw designs.

A further impetus to WA was given by the production of thermoplastic starch (TPS). Starch can be converted into a thermoplastic product under action of water/plasticizer during melt compounding. The presence of water is vital for the destructurization/gelatinization of starch. Water is usually completely evaporated during compounding and only the plasticizer remains in the TPS compound. Considering this process, the following question arises: Is it possible to produce nanocomposites by using a surfactant- or plasticizer-based masterbatch (MB) of the nanofiller? The related process versions are called surfactant- and plasticizer-assisted melt compounding procedures, respectively. The feasibility of the former one has been recently demonstrated by Hassinger *et al.* [29]. The authors used polysorbate as liquid dispersing agent for Al_2_O_3_ and TiO_2_ nanofillers, which were then directly incorporated into PA6 in a common injection molding process. The dispersion quality of the nanofillers was better when they were dispersed initially in the surfactant polysorbate. The polysorbate remained in the PS-6 and worked as plasticizer. Such techniques are expected to be developed for the nanomodification of high temperature resistant thermoplastic matrices. The fact behind this suggestion is that surfactants, dispersing aids or even solvents, suitable for the dispersion of nanofillers should have compatibility, suitable boiling point and low vapor tension, which all can hardly be met simultaneously, if at all. Therefore these carrier fluids, at least partly, always remain and act as plasticizers in the corresponding nanocomposites. Recall that the method of Hassinger *et al.* [29] conforms with “direct processing”.

It is obvious that not all thermoplastic polymers are suitable matrices for the production of nanocomposites via WA technique. Those that are susceptible to hydrolytic, hygrothermal degradation (PAs and especially polyesters) require attentions. Interestingly, PAs do not degrade markedly, as shown later. A possible reason behind this fact is that the amide groups are quite resistant to hygrothermal degradation at the processing conditions (relatively low temperatures and short residence times). Unlike PAs, polyesters, such as polyethylene terephthalates (PETs) undergo a prominent hydrolytic degradation that should be taken into account. Restoration of the MW reduction may happen *in situ* using low MW coreactive chain extenders (e.g., bi- and multifunctional epoxy resins) or after processing through solid state polymerization (SSP).

Next we shall give an overview on the achievements with WA techniques thereby distinguishing whether the water or the aqueous liquid is fully or partly evaporated during processing. The tabular listing informs the reader about the base polymer, type and amount of the nanofiller, further additives when used, compounding characteristics, major results and basic outcome of the cited works.

## 4. WA Melt Compounding of Thermoplastic Nanocomposites

Because all works so far addressed either commodity or engineering thermoplastics, this classification will be next.

### 4.1. Commodity Thermoplastics

The related works are summarized in Table 1.

Results listed in Table 1 clearly demonstrate that WA melt compounding with pristine clays is as efficient as the incorporation of suitable organoclays. Interestingly, this technique has not yet been adapted for polyolefin-containing blends though many of them are of great practical importance. Moreover, nanofillers may act as phase compatibilizers, stabilizers in polymer blends [31], which may be an interesting target for WA melt compounding in the near future. On the other hand, WA cannot convert micro- to nanofibrillated cellulose [27], *i.e.*, not capable of replacing the usual nanofibrillation techniques (like steam explosion). Incorporation of suitable rubber lattices together with nanofillers via WA into PP or PS may open a new horizon to produce toughened nanocomposites.

**Table 1:** Water-assisted (WA) melt compounding of commodity thermoplastics.

Polymer	Filler Type, Amount	Surfactant Type, Amount	Compatibilizer, toughener type, amount	Compounding	Results	Ref.
LDPE granule/powder	Microcrystalline cellulose 0–30 wt%	-	-	Water injected in the high-pressure compression zone (~125 bar) of a corotating twin-screw extruder.	Cellulose could not be fibrillated in nanoscale. WA contributed to a better dispersion of cellulose compared to the reference “dry” process.	[32]
LDPE LLDPE	Na-MMT 0–5 wt%	Various quaternary ammonium salts	LDPE-g-MA 0–10 wt%	Water, clay slurry, or aqueous surfactant were injected in the high pressure zone of an intermeshing twin-screw extruder (Figure 5).	Design of experiments used to determine effects of surfactants (type, amount) clay amount and processing conditions on mechanical, rheological and barrier properties.	[30,33]
PP 70, 100 part	Na-MMT 0, 5, 10 part	Octadecyl trimethyl ammonium chloride 0, 0.25, 1 part	PP-g-MA 0, 30 part	Corotating intermeshing extruder of very high length-to-diameter ratio (L/D = 77) and special screw design and sealings against high pressure used. Clay slurry injected.	PP/clay nanocomposite by WA melt compounding exhibited similar properties as the reference PP/organoclay. Polymeric compatibilizer (PP-g-MA) required to support MMT exfoliation.	[34]
PP PP-g-MA	Na-MMT, organoclay 21 wt%	-	-	Water injected (amount varied) in the high-pressure compression zone of a corotating twin-screw extruder. PP/(organo)clay masterbatches (MB) also processed by WA technique.	Morphological, mechanical, rheological and thermal properties of the nanocomposites studied. The MB process outperformed the “one pot” version. Water improved the dispersion of clay and proved beneficial to support the chemical reaction between PP-g-MA and hydroxyl groups of the organoclay surfactant.	[35]
PP	Na-MMT, organoclay <7 wt%	-	PP-g-MA (9–10 wt%), Na-acetate (0, 4 wt%) (to convert PP-g-MA into an ionomer)	Water injected in the high-pressure compression zone of a corotating twin-screw extruder.	Morphological, mechanical, rheological and thermal properties assessed. *In situ* synthesis of “carboxylate clay” from pristine clay and PP-g-MA ionomer, through trihydrate sodium acetate addition with help of WA compounding proved to be an effective alternative to using organomodified clays and compatibilizers.	[36]
TPV (PP-based)	CNF aqueous dispersion (15 g/L) 5 phr	-	EPDM	Crumb EPDM was spray-coated by CNF and melt mixed with TPV.	Morphology, dynamic-mechanical, thermal and tribological properties determined. The fragmented CNF was located in the PP phase.	[37]
TPV (PP-based)	BA (particle size in water 300 nm) 5 wt%	-	-	BA added dry or via WA technique using a corotating twin-screw extruder.	Tensile, thermal, DMA, creep and stress relaxation tests performed. BA located in the PP-phase. WA produced better dispersion than the traditional dry dosage. The better dispersion was best reflected in the creep and stress relaxation results.	[38]
PS	Na-fluorohectorite 0–7 wt%	-	-	Micro- and nanocomposites produced batchwise in a kneader. For nanocomposite preparation Na-fluorohectorite was mixed first with a PS latex which after drying was used as a MB for dilution with molten PS. Dry melt mixing, resulted in microcomposite.	Nanocomposites outperformed the microcomposites with respect to stiffness and resistance to creep. Dispersion in nanoscale affected, however, mostly the initial creep compliance.	[39,40]
PS	BA (particle size in water 25 and 220 nm, respectively) 4.5 wt%	-	-	Nanocomposites produced batchwise in a kneader; dry or through WA technique (latex-mediated). In the latter case PS latex was compounded with BA followed by drying and dilution with molten PS.	Latex-mediated nanocomposites exhibited higher stiffness, resistance to creep, to thermal deflection than the reference composite produced by traditional “dry” melt compounding.	[41]
PS	BA (particle size in water 25 and 220 nm, respectively) 3 wt%	-	SBR from latex 10 wt%	Binary (PS/BA, PS/SBR) and ternary systems (PS/BA/SBR) were produced via WA in a twin-screw extruder	Morphology, DMA, tensile mechanical, impact and short term creep and stress relaxation behaviors studied. BA acted as efficient nanoreinforcement while SBR as toughening agent in the binary systems. BA was mostly embedded in the SBR phase in the ternary blends. Modifiers’ effects best manifested in tensile and stress relaxation tests.	[42]

**Figure 5:**
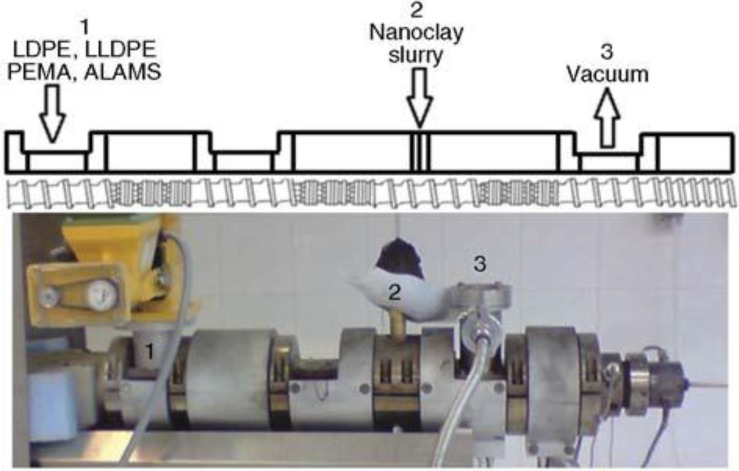
Schematic configuration of the screw and the order of mixing of the materials (**top**) and the actual twin-screw extruder during sample preparation process (**bottom**) [30,33] (with permission of BME-PT) [30].

### 4.2. Engineering Thermoplastics

Based on Table 2 the following conclusions can be drawn:

- Pristine MMT (Na-MMT) and similar cationic clays can be well dispersed in different PAs, including PA-based elastomers. The hydrolytic degradation of the PAs is small to negligible with increasing methylene groups in the structural unit. The water-induced cryoscopic effect lowers the actual viscosity and thus enhances the molecular mobility of the PA. This, along with the improved hydrophilic character of the PA chains strongly support the intercalation, and even result in full exfoliation of the clay.
- WA is less efficient for apolar polymers in absence of suitable compatibilizers. Recall that this is the learning from studies performed on polyolefins, as well.
- In case of thermoplastic polyesters, precautions are needed to avoid/compensate the prominent hygrothermal degradation manifesting in highly reduced average MW.
- The promise of WA technique is not yet explored for carbonaceous nanofillers and polymer blends. For the latter it seems to be very promising to disperse these carbonaceous nanofillers in water-soluble polymers and incorporate the related concentrated MBs in engineering polymers.

**Table 2:** Water-assisted (WA) melt compounding of engineering thermoplastics.

Polymer	Filler type, amount	Surfactant Type, Amount	Compatibilizer, toughener type, amount	Compounding	Results	Ref.
PA-6	Na-MMT 1.6 wt%	-	-	Aqueous clay slurry injected in the high-pressure zone of a corotating extruder equipped with a sealing zone.	Clay dispersion, mechanical and barrier properties determined and compared with the effect of an organoclay (stearyl ammonium ion) melt compounded “dry”. The properties were practically the same. WA only slightly reduced the MW of PA-6.	[26]
PA-6	Na-MMT nano-ZnO	octadecyl ammonium salt	epoxy resin (EP)	Compounding in a twin-screw extruder but not disclosing how water and other additives were introduced.	EP supposed to enter into the galleries and react with the terminal groups of PA-6. Incorporation of ZnO contributed to better intercalation of MMT, the reason of which was unknown. According to our feeling, this may be linked with coordination complexing between the Zn^2+^ and amid groups of PA-6 [43].	[44]
PA-6	Na-MMT organoMMT (dioctadecyl dime-thylammonium ion), 5 wt%	-	-	Compounding on a twin-screw extruder water injected into the extruder barrel downstream at various flow rates.	Morphology, mechanical, tribological and thermal properties determined. The hydrolysis of PA-6 was negligible. Unlike to organoMMT, WA compounding strongly improved the dispersion and reinforcing effectiveness of Na-MMT.	[45,46]
PA-6	Na-MMT	-	-	Water is pumped into the high-pressure compression zone of the twin-screw extruder with special screw design.	Morphology studied, cryoscopic effect of water emphasized (*cf.* Figure 6). Model proposed for the exfoliation of pristine clay, *cf.* Figure 2.	[47,48]
PA-6	Na-fluorohectorite BA (mean size in water dispersion 220 nm) 2.5 wt%	-	HNBR from latex 9 wt%	Binary (PA-6/nanofiller) and ternary systems (PA-6/nanofiller/HNBR) were produced in a kneader via WA. In the aqueous HNBR latex were also the nanofillers dispersed.	Morphology, tensile, impact, DMA and creep properties determined. Na-fluorohectorite was embedded in the PA-6 matrix, whereas BA into the dispersed HNBR domains. HNBR acted as efficient impact modifier. Na- fluorohectorite outperformed BA with respect to the properties tested. This was traced to its preferred dispersion in the PA-6 matrix.	[49,50]
PA-6	Na-MMT 1.5, 3 wt%	-	-	Two-step extrusion process used. First step: MB production with and without WA. Second step: dilution of MB with and without WA. Water injected at >26 bar in the mixing zone of the extruder.	High level of MMT, reflected in the mechanical properties, achieved with longer contact time between water and PA-6 melt. Accordingly, the WA process is controlled by diffusion mechanism.	[51]
PA-6/PP blend (PP major phase)	Na-MMT	-	SEBS-g-MA	PA/clay (60/20 wt%) nanocomposite produced by WA melt compounding and it was used as MB to dilute with PP.	The compatibilizer (SEBS-g-MA) was located in the interphase between PA-6 (in submicron nodules) and PP matrix.	[48]
PA-11	Na-MMT 0–20 wt%	-	-	Water pumped into the high-pressure compression zone of a twin-screw extruder of special design.	Exfoliated morphology demonstrated up to 10 wt% clay. Stiffness and thermal stability of PA-11 are drastically enhanced, ductility decreased. Based on WAXS the crystal axis was parallel to the clay surface. Strong effect of screw rotation speed concluded.	[52]
PA-12	Halloysite 0–16 wt%	-	-	Water injected into the high-pressure compression zone (~125 bar) of the twin-screw extruder.	Fracture, tensile, thermal and flammability properties determined. Stiffness, strength markedly improved at cost of elongation at break. Water was an efficient dispersing aid for halloysite. Improved dispersion ascribed to potential H-bond formation between PA-12 and surface hydroxyl groups of halloysite.	[53]
PEBA	raw MMT (non-purified bentonite) Na-MMT organoMMT	-	-	Water injected into the high-pressure zone (70–100 bar) of the twin-screw extruder. Pressure of the injected water higher than the water vapor pressure at the processing temperature.	PEBA degradation checked by GPC and no hydrolytic degradation found. Clay dispersion, morphological, tensile properties determined. Stiffness, strength strongly enhanced at cost of ductility with increasing MMT content. Properties comparable with compounds with organoMMT.	[54]
PET	MMT centrifuged MMT 0–2 wt%	-	-	Clay slurry (through a peristaltic pump) and PET granules fed into a corotating twin-screw extruder.	Morphology and melt viscosity determined. Centrifuged clay (having no large agglomerates) yielded better dispersion than MMT.	[55]
PET	Na-MMT organoMMT (different surfactants) 0–6 wt%	-	-	PET with dry mixed clay was fed into the extruder. Water steam (160 °C saturated or not) was introduced in the second zone of a corotating twin-screw extruder with special screw design.	MW degradation determined by measuring the intrinsic viscosity. MW markedly decreased by WA compounding, reflected by a large drop in the ductility. WA method resulted in better stiffness, strength than traditional “dry” one. To compensate MW degradation, solid-state polymerization (SSP) was performed. No improvement of SSP was found at high organoMMT contents. Rheological results proved to be useful indicators of the clay dispersion.	[56,57,58]
SAN	Na-MMT organoMMT 0–3 wt%	-	-	Water pumped into the high-pressure compression zone (~125 bar).	Changes in MW and morphology determined. Dynamic-mechanical analysis, mechanical and flammability tests conducted. WA improved the dispersion of organoMMT and Na-MMT. According to XRD no intercalation was observed for Na-MMT.	[59]
POM	BA in different nanodimensions 3 wt%	-	-	Aqueous BA slurry introduced in the low-pressure feeding zone of a twin-screw extruder.	BA dispersion with its effects on DMA and creep properties studied. BA of smaller size resulted in better property improvement than the coarser one.	[60]
POM	BA 0, 3 wt%	-	PU (from latex) 0, 10 wt%	Binary (POM/PU, POM/BA) and ternary (POM/PU/BA) systems produced by WA method. PU latex and aqueous BA slurry introduced in the low pressure feeding zone of a twin-screw extruder.	Morphology, DMA, creep, tensile and impact properties determined. Good dispersion in the binary system, BA embedded in the PU in the ternary nanocomposite, *cf.* Figure 7.	[28]
POM	CNF 0.1 wt%	-	PU (from latex) 0, 10 wt%	Binary (POM/PU, POM/CNF) and ternary (POM/PU/CNF) systems produced by WA in a kneader (inner mixer)	Morphology, crystallinity, DMA, creep, stress relaxation and dielectrical properties studied. CNF worked as nanoreinforcement.	[61]

**Figure 6:**
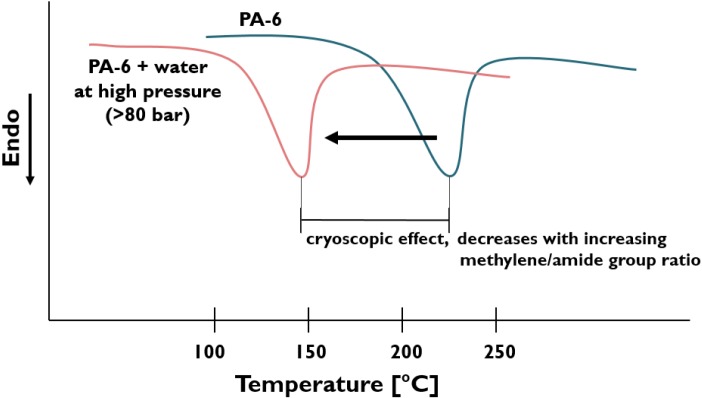
Differential scanning calorimetric (DSC) thermogram of polyamide 6 (PA-6) and PA-6 with water [47,48].

**Figure 7:**
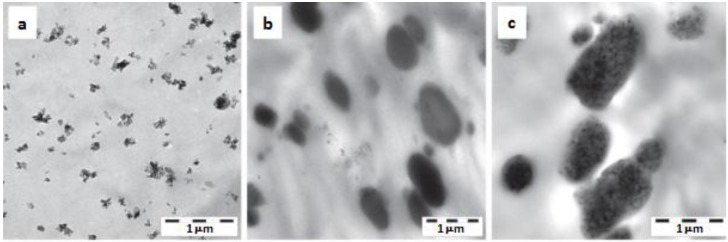
Dispersion of (**a**) boehmite alumina (BA); (**b** polyurethane (PU)); (**c**) BA + PU in a polyoxymethylene (POM) based system based on transmission electron microscopy (TEM) investigations [28]. Note (**a**) the fine dispersion of BA; (**b**) the micron range dispersion of PU (the usual requirement for impact modifier); (**c**) the BA embedding in the PU phase.

## 5. WA Melt Compounding of Plasticized Thermoplastic Nanocomposites

As quoted before, this strategy was fuelled by the production of thermoplastic starch (TPS). Native starch granules swell by water absorption through H-bonding, in which the free hydroxyl groups participate. Nevertheless, the crystalline order still remains well detectable. The crystalline structure of TPS can be destroyed and the H-bonds disrupted under action of heat and pressure. This yields gelatinized starch. Addition of plasticizers can improve the properties and especially the ductility of gelatinized starch. Since the related material is capable of flowing, it is called TPS. Starch suspensions with plasticizer and water are converted to TPS in one-step extrusion. TPS can be modified in line with different polymers. In line blending with LDPE was practiced by Rodriguez-Gonzalez *et al.* [62]. Taguet *et al.* [63] produced HDPE/TPS (80 wt%/20 wt%) blends in one-step extrusion. The extrusion system was composed of a single-screw extruder (feeding HDPE and HDPE-g-MA) connected midway to a corotating twin-screw extruder. The later was fed by the starch/water/glycerol suspension. As expected, the TPS size was reduced in the blend by the compatibilizer, but it was associated with a drop in the fracture resistance. This was traced to the reaction between the maleic anhydride and hydroxyl groups of the glycerol leading to a decrease of the plasticizer content. In order to keep the green character of TPS, it seemed to be more appropriate to blend TPS with biobased and/or biodegradable polymers, such as polylactic acid (PLA). Recall that TPS itself with a low amount of plasticizer is very brittle. Huneault and Li [64] reported on compatibilized TPS/PLA blends, produced in a similar way as disclosed in Ref. [62]. Compatibilization in this case means that PLA has been previously or *in situ* grafted with MA (PLA-g-MA) in a peroxide initiated grafting process. This compatibilization strategy enhanced the elongation at break values for 100% or more, which is a prerequisite for biodegradable packaging applications. In a companion work, the same authors studied the effects of plasticizers, namely sorbitol and glycerol, and their combinations. It was found that the sorbitol/glycerol ratio has a prominent effect of the PLA(matrix)/TPS(dispersed phase) blends [65]. Effects of the type and amount of the plasticizers on the TPS performance were emphasized by Mikus *et al.*, as well [66]. Researchers soon recognized the importance of the results achieved with the WA melt incorporation of natural clays (*cf.* Section 4) and adapted it to TPS-based systems. It was found that incorporation of 5 wt% Na-MMT enhanced the E-modulus of glycerol plasticized cast starch film up to 500% and reduced its water uptake at the same time [67]. Arroyo *et al.* [68] produced MMT filled (up to 5 wt%) TPS in extrusion compounding, during which the extruder was fed with a starch and MMT containing slurry. Because nanofillers *per se* decrease the ductility of the related nanocomposites, PLA or PLA-g-MA was in line incorporated. Clay particles were embedded in the TPS phase. By contrast, when clay was introduced together with PLA, then it migrated into the interphase between TPS and PLA. This strongly affected the fracture behavior of the resulting nanocomposites. This is the right place to mention that WA may contribute to odor emission reduction in cellulose containing compounds, which is of great relevance for automotive applications [69]. A straightforward strategy has been proposed recently by Hietala *et al.* [70]. The cited authors first produced an aqueous cellulose nanofiber suspension by the usual way. This suspension was fed into the extruder along with starch, water and plasticizer in order to prepare nanocellulose reinforced TPS. The beauty of this method is that nanocellulose was incorporated in aqueous suspension. Nanocellulose in dry form can hardly be disintegrated in traditional melt compounding because the fibrils are entangled and strongly held together by H-bonding. TPS was modified with up to 20 wt% nanocellulose. This modification doubled the tensile strength, caused a threefold increase in the E-modulus, but was accompanied with a prominent drop in the elongation at break (from the initial 23% of TPS to 2% in the TPS nanocomposite with 20 wt% nanocellulose). Our group followed a similar approach. Microcellulose filled TPS composite systems were produced by the WA technique using a twin-screw extruder. Glycerol was used as plasticizer. The cellulose fibers, dispersed in water, were introduced into the extruder. After granulating the extrudates, specimens were produced by a compression molding and subjected to tensile tests. The related results are summarized in Table 3.

**Table 3:** Mechanical properties of thermoplastic starch (TPS) based composite systems filled with different amount of microfibrillated cellulose.

Microcellulose content (wt%)	NR Latex content (wt%)	Young’s modulus (GPa)	Yield strength (MPa)	Elongation at yield (%)
5	-	0.15 ± 0.02	4.7 ± 0.7	39.3 ± 5.8
10	-	0.16 ± 0.02	4.9 ± 0.3	26.9 ± 2.7
10	10	0.31 ± 0.03	3.1 ± 0.4	2.2 ± 0.1
15	-	0.17 ± 0.01	5.3 ± 0.3	19.6 ± 1.8
20	-	0.23 ± 0.04	5.6 ± 0.7	15.3 ± 0.9
20	10	0.53 ± 0.05	3.1 ± 0.4	1.0 ± 0.3

**Figure 8:**
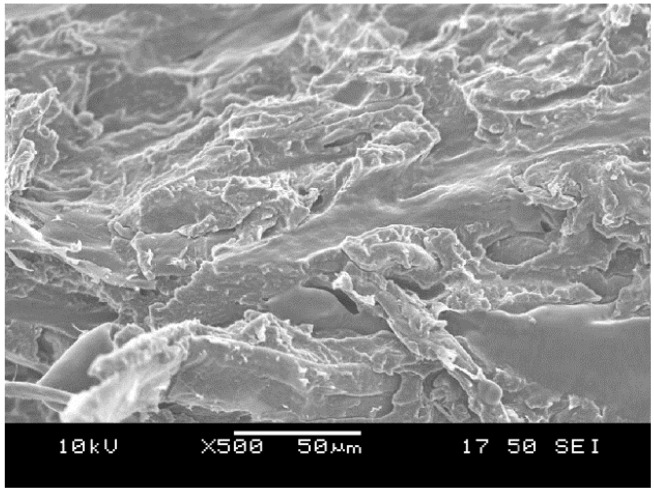
Scanning electron microscopy (SEM) picture taken from the fracture surface of a thermoplastic starch (TPS) containing microfibrillar cellulose (10 wt%) and natural rubber (NR) (10 wt%). The deformed NR particles’ surface is smooth.

Recognizing the strong ductility decrease with increasing amount of microfibrillated cellulose (*cf.* Table 3), attempt was made to improve the ductility of TPS. For this purpose the “latex concept” was followed and NR latex was added during production of the TPS and its cellulosic nanocomposites *cf.* Table 3. Contrary to our expectations, it resulted in a further drop of the elongation at yield, however, the E-modulus increased significantly, implying the presence of a hybrid effect. This finding may be interpreted by considering that the modulus of NR is higher than that of TPS, and NR partly encapsulates the cellulosic particles. Figure 8 seems to support this explanation.

Modification of TPS and its blends with nanofillers is a highly actual topic [71]. Beside of improvements in the mechanical property profile (especially ductility and thus toughness), further potential targets are barrier properties, and even some functional ones. Recall that by water uptake the T_g_ of TPS can be reduced. This can be exploited for the preparation of humidity sensitive one-way shape memory polymers [72,73]. Nanocellulose efficiently decreased the moisture content of TPS at equilibrium [74] and strongly improved the barrier resistance of PLA [75]. Further trials will be done to improve the dispersion of cellulosic nanofillers, cationic (both pristine and organoclays) and anionic clays (only surface modified versions [76]), and especially halloysite nanotubes as proposed by Schmitt *et al.* [77].

## 6. Conclusions and Outlook

Water-assisted melt compounding remains under spot of research interest further on [78]. This is due to the fact that the properties of nanocomposites produced by WA are comparable with those using organophilic-modified surface treated nanofillers. Moreover, WA, or more exactly, liquid-assisted melt compounding opens a new horizon in the production of plasticized thermoplastic nanocomposites.

Because practically no information is available on how a conventional extruder should be adjusted for the WA technique, this aspect will be topic of extensive investigations. Accordingly, future works will focus on processing, *i.e.*, on optimizing the extruder parameters (screw configuration along with the pressure profile, temperatures, residence time, *etc*.). It is still an open question whether introduction of water should occur in the feeding or in the compression zone of the extruder. In order to improve its efficiency, different water-soluble additives, promoting the dispersion and surface modification of the corresponding nanofillers (salts, surfactants, thickeners) will be used. In this respect research in the near future will focus exploring the use of ionic [79,80] and eutectic liquids as dispersion aids of the nanofillers. Types of the nanofillers will likely remain at the introduced ones. Vivid research and development works are expected for the production of high temperature resistant thermoplastic nanocomposites. Here new concepts will be elaborated and their feasibility checked. Instead of water, however, other fluids (with adjusted boiling point and vapor pressure), monomers and polymers, which may overtake further roles (e.g., chain coupling, reactive surface modification, suspension stabilizer), will be preferentially addressed. Further potential application of TPS-based systems (including nanocomposites) in packaging, such as biodegradable cushioning materials, will fuel the research on their foaming thereby using water as blowing agent [77,81].

## List of Abbreviations

0D: zero-dimensional
1D: one-dimensional
2D: two-dimensional
3D: three-dimensional
ALAMS: alkyl ammonium salt
Al_2_O_3_: aluminum oxide
AlO(OH): aluminum oxide hydroxide
BA: boehmite alumina
DW-CNT: double-walled carbon nanotube
Ca: calcium
CNF: carbon nanofiber
CNT: carbon nanotube
DMA: dynamic mechanical analysis
DSC: differential scanning calorimetry
EPDM: ethylene propylene diene rubber
EVA: ethylene vinylacetate copolymer
GPC: gel permeation chromatography
H: hydrogen
HDPE: high density polyethylene
HNBR: hydrogenated nitrile (acrylonitrile butadiene) rubber
LDPE: low density polyethylene
LDPE-g-MA: maleated low-density polyethylene (grafted by maleic anhydride)
LLDPE: linear low density polyethylene
K: potassium
MA: maleic anhydride
MB: masterbatch
MMT: montmorillonite
MW: molecular weight
MW-CNT: multi-walled carbon nanotube
Na: sodium
Na-MMT: sodium montmorillonite
NR: natural rubber
organoMMT: organophilic modified montmorillonite
OTAC: octadecyl trimethyl ammonium chloride
PA: polyamide
PE: polyethylene
PEBA: polyether-block-amide
PEMA: polyethyl methacrylate
PET: polyethylene terephthalate
PLA: polylactic acid
PLA-g-MA: maleated polylactic acid (grafted by maleic anhydride)
POM: polyoxymethylene
PP: polypropylene
PP-g-MA: maleated polypropylene (grafted by maleic anhydride)
PS: polystyrene
PU: polyurethane
SAN: styrene acrylonitril copolymer
SBR: styrene butadiene rubber
SEBS-g-MA: maleated styrene-ethylene butylene-styrene block copolymer
SEM: scanning electron microscopy
SSP: solid state polymerization
SW-CNT: single-walled carbon nanotube
TEM: transmission electron microscopy
TPS: thermoplastic starch
TPV: thermoplastic vulcanizate
TiO_2_: titanium dioxide
WA: water-assisted
XRD: X-ray diffraction
ZnO: zinc oxide
